# The p. N103K mutation of leptin (*LEP*) gene and severe early onset obesity in Pakistan

**DOI:** 10.1186/s40659-016-0082-7

**Published:** 2016-04-13

**Authors:** Shabana, Shahida Hasnain

**Affiliations:** Department of Microbiology and Molecular Genetics, University of the Punjab, Lahore, 54590 Pakistan; The Women University Multan, Multan, Pakistan

**Keywords:** Obesity, Leptin, p. N103K, Pakistan

## Abstract

**Background:**

Obesity is a complex disorder and has been increasing globally at alarming rates including Pakistan. However, there is scarce research on understanding obesity genetics in Pakistan. Leptin is a hormone secreted by adipocytes in response to satiety and correlates with body weight. Any mutations in the *LEP* gene have an adverse effect on energy regulation pathway and lead to severe, early onset obesity. To date, only eight mutations have been described in the *LEP* gene of which p. N103K is one.

**Methods:**

We aimed to analyze the prevalence of this mutation in Pakistani subjects. A total of 475 subjects were genotyped by PCR–RFLP analysis and their serum profiling was done.

**Results:**

Results showed that this mutation was present only in one male child with early onset obesity (10 year). He had very low serum leptin levels suggestive of functional impact of the mutation. The prevalence of such mutations is, however, low due to the drastic effects on the energy regulation.

**Conclusion:**

In conclusion, *LEP* gene mutations contribute significantly to the monogenic forms of obesity and are important due to the availability of treatment options. Such mutations may exert their effect by directly affecting energy regulation pathway and are more prominent in the early stages of life only.

## Background

Obesity is defined as a medical condition in which excess body fat accumulation can impact health negatively. It has become one of the leading disorders afflicting mankind worldwide. Due to recent explosion of the obesity World Health Organization (WHO) has designated obesity a global epidemic. Obesity is a heterogeneous disorder involving a complex interaction of multitude of including environmental, behavioural and genetic factors, none of which are completely understood [1]. Genetic studies on obesity started as a result of the observation that family clustering occurs in obesity. A landmark discovery in the field of obesity genetics was the identification of mutations in the leptin gene in grossly obese children followed by identification of mutations in other genes involved in energy regulation pathways laying down the basis for monogenic obesity [2].

Leptin is a protein secreted by adipocytes, the fat cells, and its concentration in blood positively correlates with body fat mass and body mass index (BMI). It has a distinct impact on various physiological processes including energy metabolism, endocrine and immune systems [3]. It controls body fat by inhibiting food intake due to targeting central nervous system. In addition to controlling body fat, leptin also has important funtions in reproductive organs, mammary glands, immune system and bone mineral density [4]. Leptin hormone is a member of long chain helical cytokines’ family, the other members of the family are important regulators e.g., interleukin-6, granulocyte colony-stimulating factor and growth hormone [5]. The hormone is a synthesized as 167 amino acid protein which is immature. The processing involves cleavage of a 21-amino acid N-terminal signal sequence producing a mature functional non glycosylated 146-amino acid protein [6]. *LEP* gene is present on 7q31.3 chromosomal region and includes three exons intervened by two introns [7]. The gene was identified in 1994 by positional cloning and is ~16 kb long [6]. Only eight mutations have been identified in *LEP* gene at the present causing severe early onset obesity [4, 8–17]. We selected g.13285C>A missense mutation originally reported in an obese Egyptian child, to investigate whether this mutation is present in Pakistani population or is restricted to a particular ethnic group. This mutation leads to substitution of asparagine to lysine at position 103 (p. N103K) at protein level [4] causing a reduction of biological activity of the mutant protein and very low serum leptin levels [18].

Pakistan with a total population of 184.35 millions in 2012–2013 is the 6th most populous country of the world. According to the Global Burden of Disease Study, in terms of obesity, it ranked 9th out of 188 countries [19]. Pakistan faced a lot of health challenges during a decade long war on terror [20]. *LEP* gene p. N103K mutation has not been investigated in the Pakistani subjects previously. Keeping in view the global research perspective, limited research with regard to obesity and no research of the mutation in Pakistan, we aimed to find out whether this mutation plays any role in obesity in the Pakistani population and which serum parameters are affected by the mutation, if there are any.

## Methods

### Study subjects

The study was designed as a case control observational type. Subjects were recruited from January 2011 to June 2014 from different areas of Punjab Pakistan. A total of 475 unrelated subjects was selected after obtaining informed consent and the subjects filled in a detailed questionnaire related to diet, lifestyle, disease and family history. The recruitmen, inclusion and exclusion criteria to define and differentiate different categories of subjects have been described elsewhere [21].

### Ethics, consent and permissions

All procedures were in compliance of Helsinki Declaration and the study was approved by the institutional ethics committee (Ethical Committee, School of Biological Sciences, University of the Punjab, Pakistan).

### Anthropometric traits’ measurement

Anthropometric traits including weight (Wt), height (Ht), waist and hip circumference (WC, HC), systolic and diastolic blood pressure (SBP, DBP) were measured as described previously [21].

### Biochemical analyses

5 ml of blood was drawn by venipuncture from the median cubital vein after 8–12 h fasting, 2 ml was poured in a gel clot activator containing vacutainer that was centrifuged to separate plasma used for determination of biochemical parameters while 3 ml was poured in an EDTA containing vacutainer that prevented blood from clotting and was used for DNA isolation. Serum was screened for HBV, HCV and HIV. Positive samples were discarded and safe samples were proceeded. Serum fasting plasma glucose (FPG), total cholesterol (TC), triglycerides (TG), High Density and Low Density lipoprotein cholesterol (HDL-c, LDL-c), were determined using commercially available kits (Spectrum Diagnostics, Egypt), leptin by LDN Nordhorn leptin ELISA kit whereas insulin concentration was measured using electrochemiluminescence method as described previously [22], and HOMA-IR was calculated.

### Genetic analysis

Genomic DNA was isolated from blood leukocytes by salting out method. The primers used for genotyping p. N103K mutation were described previously [23]. The sequence of the primers was forward: 5′-GCACTTGTTCTCCCTCTTCCT-3′ and reverse:, 5′-GTTCCTTCCCTTAACGTAGTCCT-3′ and were synthesized by Gene Link™, USA. PCR reaction conditions consisted of initial denaturation at 95 °C for 2 min, 35 cycles of denaturation at 95 °C for 35 s, annealing at 53 °C for 30 s, then extension at 72 °C for 30 s and a final extension at 72 °C for 5 min. The 438 bp PCR product contains seven restriction sites for *Mnl*I in the wild type state. Presence of mutation leads to the loss of one of the seven restriction sites. 5 µl of the PCR product was used for digestion with *Mnl*I (Thermo Fisher Scientific, USA). The digestion mixture containing PCR product, enzyme and an appropriate enzyme buffer was incubated at 37 °C for 6 h.

## Results

The characteristics of the study subjects included in the study have been described previously [21]. All parameters except height differed significantly between cases and controls as tested by independent sample *t* test. The FBG, systolic and diastolic blood pressure, leptin, insulin and HOMA-IR were significantly elevated in obese cases as compared to controls while the lipid profile was also dyslipidemic in the case group.

**Fig. 1:**
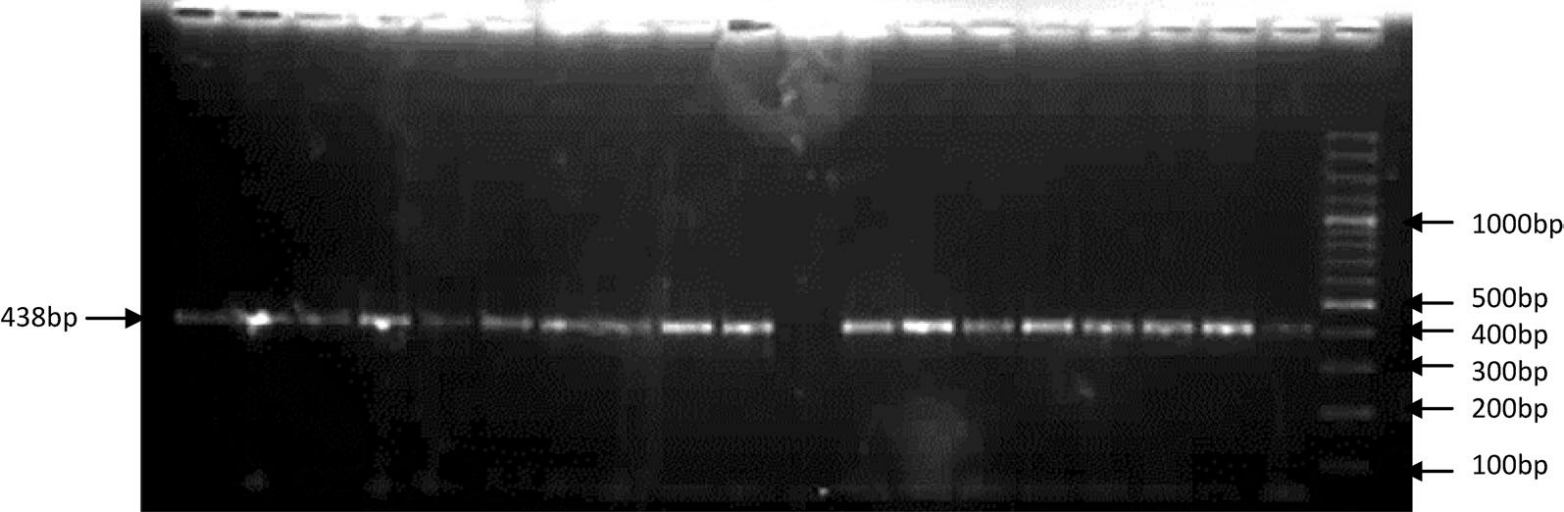
Amplification of the exon 3 of the leptin gene containing N103K mutation

**Fig. 2:**
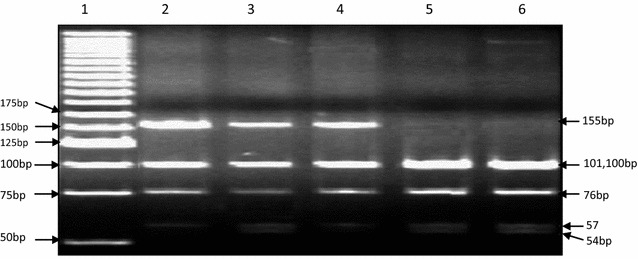
Polyacrylamide gel electrophoresis of restriction digestion of amplified region for N103K, *lane 1* is marker (25 bp DNA ladder SM#1191, Thermo Scientific), *lanes 2 and 4* are heterozygous parents, *lane 3* is homozygous proband, and *lanes 5, 6* are controls. Presence of mutation abolishes one of the seven restriction sites of MnlI

**Fig. 3:**
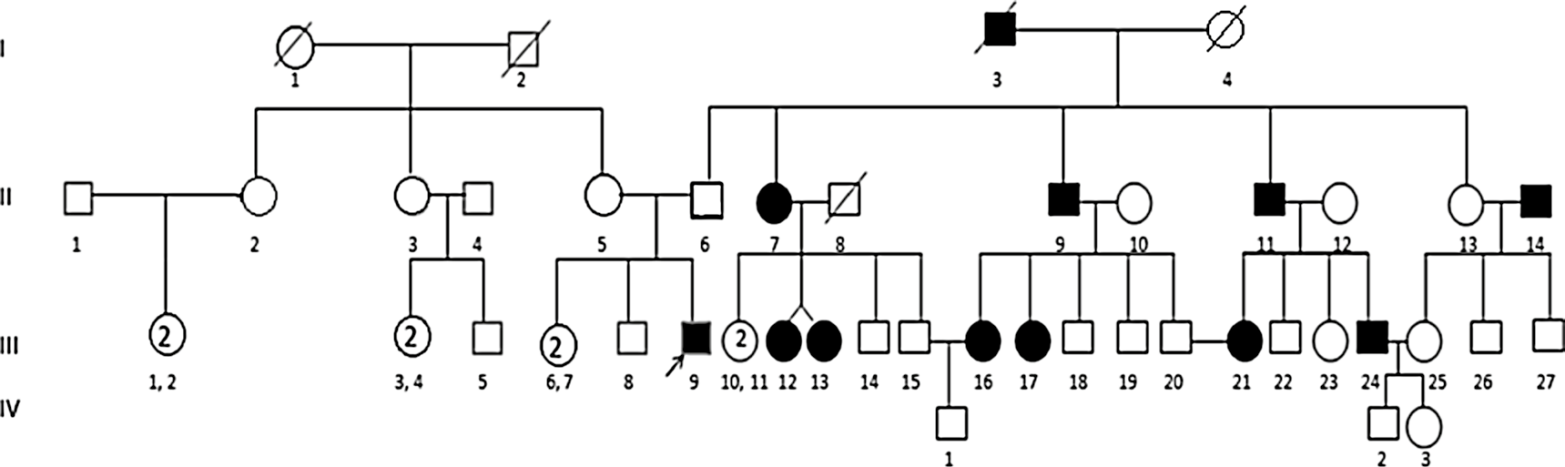
Pedigree of the Propositus. *I-1*: Proband’s deceased maternal grandmother. *I-2*: Proband’s deceased maternal grandfather. *I-3*: Proband’s deceased paternal grandfather (reported as obese by proband’s father, genotype not available). *I-4*: Proband’s deceased paternal grandmother (reported as overweight by proband’s father, genotype not available). *II-5*: Proband’s normal weight mother (BMI: 23.9 kg/m^2^, genotype CA). *II-6*: Proband’s normal weight mother (BMI: 26.5 kg/m^2^, genotype CA). *III-6, 7*: Proband’s normal weight sisters (BMI < 22 kg/m^2^, genotype CC). *III-8*: Proband’s overweight elder brother (BMI: 27.2 kg/m^2^, genotype CA). *III-9*: Proband, age of onset: 10 (BMI: 48.44 kg/m^2^, genotype AA)

We could detect p. N103K mutation in homozygous state in only one subject (Figs. 1, 2). When the history of the subject was studied, it was a boy with age 10 years, early onset of obesity, hyperphagia, weighed 85 kg with height 85 cm (BMI = 48.44 kg/m^2^), other siblings included two normal weight sisters and an overweight elder brother, parents were normal weight (BMI of mother was 23.9 kg/m^2^ and of father 26.5 kg/m^2^), the overweight brother and parents were found to be heterozygous for the mutation while the child had p. N103K mutation in homozygous state. The family was a large, highly consanguineous one with multiple obese individuals (Fig. 3). The serum lipid profile and leptin levels of all study subjects were determined. Lipid profile of the propositus turned out to be in normal range, whereas leptin levels were close to the detection limit as measured by ELISA (0.1–1.0 ng/ml). Although we obtained samples from proband and his parents, other obese relatives did not agree to give blood samples therefore the inheritance pattern of the mutation could not be established. In addition, limited information was available on the maternal family history of the child with more recall bias.

## Discussion

In the current study, the prevalence of the p. N103K mutation in the coding region of the *LEP* gene was checked to demonstrate the role of *LEP* gene variations in the development of obesity in Pakistan. *LEP* gene is a known candidate gene, mutations in which contribute to severe early onset obesity due to the important physiological roles leptin performs in circulation, by ultimately affecting hypothalamus. It is a known monogenic cause of obesity worldwide and all mutations in the gene reported so far result in severe disturbance of body weight regulation (Table 1).

**Table 1:** Overview of Mutations reported in ***LEP*** **gene**

Name of mutation	Nature of change	Amino acid change	Ethnicity	Consanguinity	No of subjects	References
g. 13374delG	Frameshift	Gly-Val	Pakistani	Yes	2	Montague et al. [16]
g13289C>T	Missense	Arg-Trp	Turkish	Yes	3	Strobel et al. [15]
g. 13398C>G	Missense	Ser-Cys	Turkmenian	Unknown	Unknown	Chekhranova et al. [24]
g. 13285C>A	Missense	Asn-Lys	Egyptian	Yes	2	Mazen et al. [23]
g. 13191T>C	Missense	Leu-Ser	Austrian	Unknown	1	Funcke et al. [25]
g. 10839_10841delTCA	Codon deletion	del Isoleu	Pakistani	Yes	1	Saeed et al. [26] Fatima et al. [27]
g. 13457_13458delCT	Codon deletion	Leu-Gly	Pakistani	Yes	1	Fatima et al. [27]
g. 13139C>T	Nonsense	Gln-STOP	Indian	Yes	1	Thakur et al. [28]

Congenital leptin deficiency is a rare form of monogenic obesity and was first reported in two cousins of Pakistani origin with severe early onset obesity who had very low serum leptin levels. These two subjects were found to be homozygous for a frame shift mutation resulting in a misfolded protein which was not secreted [16]. A second mutation was identified in a Turkish family with severe early onset obesity [15]. p. N103K was the third to be identified in an Egyptian child [23]. Five more mutations were discovered afterwards in the *LEP* gene. All of these known mutations with important points are summarized in Table 2.

**Table 2:** Characteristics of the **p. N103K** homozygous subject

Trait	Observed value
Age of onset (years)	10
Weight (kg)	85
Height (cm)	85
BMI (kg/m^2^)	44.48
WC (cm)	94.55
HC (cm)	99.51
WHR (WC/HC)	0.91
FPG (mmol/l)	85.56
Total Cholesterol (TC) (mmol/l)	4.00
Triglycerides (TG) (mmol/l)	1.52
HDL-c (mmol/l)	2.39
LDL-c (mmol/l)	2.62
SBP (mmHg)	111.02
DBP (mmHg)	71.15
Leptin (ng/ml)	0.9
Insulin (µU/ml)	19.05
HOMA-IR	4.09

The mutation p. N103K in exon 3 of *LEP* gene was shown to be invariably associated very strongly with serum leptin levels. The effect of this missense mutation involves a change of asparagine to lysine at position 103 in the final protein resulting in very low serum leptin levels as observed in the current study as well as the original report of two Egyptian patients [4]. Although the effect of this amino acid change on the synthesis, secretion and biological activity of leptin was not clearly demonstrated, the assessment of altered biological activity in vitro was checked in a study by Niv-Spector et al. [29]. In this study, a prokaryotic expression system was used to produce p. N103K leptin and it was shown that this results in a drastic reduction in biological activity of the mutant leptin protein. This observation led to the hypothesis that defective biological activity in addition to already lower serum levels contributes to the severe obese phenotype seen in p. N103K patients.

## Conclusion

In conclusion, the congenital leptin deficiency is very rare and the frequency of mutations reported in *LEP* gene is very low. However, the molecular diagnosis of these mutations is very important due to the availability of treatment with recombinant leptin. The p. N103K mutation was previously reported in Egyptian child, however its presence in Pakistani group, even at very low frequency, indicates that this mutation can play important role in the development of morbid severe early-onset obesity.
